# Economic value of narrow-band imaging versus white light endoscopy for the diagnosis and surveillance of Barrett’s esophagus: Cost-consequence model

**DOI:** 10.1371/journal.pone.0212916

**Published:** 2019-03-13

**Authors:** Gianluca Furneri, Romy Klausnitzer, Laura Haycock, Zenichi Ihara

**Affiliations:** 1 Value, Access and Pricing, CBPartners, London, United Kingdom; 2 Medical Systems Division, Olympus Europa, Hamburg, Germany; Massachusetts General Hospital, UNITED STATES

## Abstract

Barrett’s esophagus (BE) is an abnormality arising from gastroesophageal reflux disease that can progressively evolve into a sequence of dysplasia and adenocarcinoma. Progression of Barrett’s esophagus into dysplasia is monitored with endoscopic surveillance. The current surveillance standard requests random biopsies plus targeted biopsies of suspicious lesions under white-light endoscopy, known as the Seattle protocol. Recently, published evidence has shown that narrow-band imaging (NBI) can guide targeted biopsies to identify dysplasia and reduce the need for random biopsies. We aimed to assess the health economic implications of adopting NBI-guided targeted biopsy vs. the Seattle protocol from a National Health Service England perspective. A decision tree model was developed to undertake a cost-consequence analysis. The model estimated total costs (i.e. staff and overheads; histopathology; adverse events; capital equipment) and clinical implications of monitoring a cohort of patients with known/suspected BE, on an annual basis. In the simulation, BE patients (N = 161,657 at Year 1; estimated annual increase: +20%) entered the model every year and underwent esophageal endoscopy. After 7 years, the adoption of NBI with targeted biopsies resulted in cost reduction of £458.0 mln vs. HD-WLE with random biopsies (overall costs: £1,966.2 mln and £2,424.2 mln, respectively). The incremental investment on capital equipment to upgrade hospitals with NBI (+£68.3 mln) was offset by savings due to the reduction of histological examinations (-£505.2 mln). Reduction of biopsies also determined savings for avoided adverse events (-£21.1 mln). In the base-case analysis, the two techniques had the same accuracy (number of correctly identified cases: 1.934 mln), but NBI was safer than HD-WLE. Budget impact analysis and cost-effectiveness analyses confirmed the findings of the cost-consequence analysis. In conclusion, NBI-guided targeted biopsies was a cost-saving strategy for NHS England, compared to current practice for detection of dysplasia in patients with BE, whilst maintaining at least comparable health outcomes for patients.

## Introduction

Barrett’s esophagus (BE) is a condition in which the typical squamous epithelium of the esophageal mucosa is replaced with columnar intestinal epithelium [1,2]. The prevalence of BE ranges from 0.5% to 2% of the general adult population, with gastroesophageal reflux disease (GERD), obesity, cigarette smoking, alcohol, and Helicobacter pylori infection being the most common risk factors of development [3].

BE can progressively evolve into a sequence of low-grade dysplasia (LG-BE), high-grade dysplasia (HG-BE), and eventually esophageal adenocarcinoma (EAC) [4], a malignancy that has witnessed increasing incidence over the past 40 years [5,6]. Large cohort studies on BE patients have reported rates of progression ranging from 0.1 to 0.3% [7–9]. The risk of EAC among patients with BE is estimated to be 30 to 125 fold greater than that of the general population [10].

Incidence of EAC is expected to continue to increase [11]. The predicted ranges of incidence and mortality rates (cases per 100,000 person years) in 2030 are 8.4 to 10.1 and 5.4 to 7.4, respectively, for males, and 1.3 to 1.8 and 0.9 to 1.2 for females, approximately doubling the number of deaths in the past 20 years, and at an accelerating rate [11].

In light of the increasing incidence and mortality burden associated with EAC, early detection of BE and surveillance of its progression into dysplasia and carcinoma are crucial clinical objectives in diagnostic endoscopy. Current guidelines recommend an endoscopic surveillance approach based on random four-quadrant biopsy specimens obtained at every 2 cm to detect dysplasia, in addition to targeted biopsies of suspicious lesions under white-light endoscopy (WLE), known as the Seattle protocol [12,13]. However, such approach may be associated with significant issues. Firstly, collection of biopsy specimens is time consuming for operators and exposes patients to a non-negligible risk of esophageal bleedings and mucosal perforation, especially in patients with long-segment BE (>3 cm in length) [14]. Secondly, diagnostic results from random biopsy can vary depending on the operator [15], with potential risk of sensitivity loss if the procedure is not performed according to protocol. Finally, interpretation of diagnostic results can be challenging and subject to inter-evaluator variance, with potential discordance among pathologists on the diagnosis of LD-BE [15].

In the last decade, several imaging techniques have been developed with the aim of overcoming the typical diagnostic challenges in BE surveillance and improving the detection of dysplasia and EAC [16].

The most well-studied, approved techniques include chromoendoscopy using acetic acid, confocal laser endomicroscopy (CLE), opto-digital chromoendoscopy with use of narrow-band imaging (NBI, Olympus) with or without autofluorescence imaging [14]. Such techniques can guide targeted biopsies for the detection of dysplasia and have the potential of eliminating or at least reducing the need for random biopsies, as shown in a recent meta-analysis [14].

More specifically, a recent study comparing NBI vs high-definition white light endoscopy (HD-WLE) has shown that NBI: i) has the same intestinal metaplasia (IM) detection rate as an HD-WLE examination with the Seattle protocol while requiring fewer biopsies; ii) can detect more areas with dysplasia than HD-WLE [17].

While the clinical implications of advanced imaging supporting BE detection has been extensively investigated (see ‘Identification of relevant clinical inputs’ for the publications identified), little is known regarding cost-effectiveness of these techniques. The aim of the present analysis is to assess the potential economic implications of adopting NBI-guided targeted biopsy for BE surveillance replacing the Seattle protocol, considered the current standard of care for screening and surveillance in BE, from a National Health Service (NHS) England perspective.

## Materials and methods

### Model structure

A combined decision tree / Markov model approach was adopted to undertake cost-consequence, budget impact, and cost-effectiveness analyses of NBI with target biopsies (hereafter, for simplicity, “NBI”) vs HD-WLE with Seattle Protocol (hereafter, for simplicity, “HD-WLE”) in the clinical management of BE from an NHS England perspective.

The decision tree approach was used to model the diagnostic and surveillance phases. At the beginning of simulation, a cohort of patients with suspected BE enter the model to undergo an endoscopic test (with either NBI or HD-WLE) and receive one of the following diagnoses: i) non-dysplastic BE (ND-BE); ii) BE with low-grade dysplasia (LG-BE); iii) BE with high-grade dysplasia (HG-BE) or EAC. HG-BE and EAC were grouped together for the sake of simplicity as the recommended surveillance intervals after treatment are identical [18]. Effectiveness of the two alternative techniques depends on their respective diagnostic accuracy.

Patients with HG-BE receive endomucosal resection (aimed at removing dysplasia) then enter the surveillance program, undergoing surveillance endoscopy every six months. Patients with EAC undergo cancer treatment, followed by surveillance endoscopy every six months. Patients with ND-BE and LG-BE do not receive any resection, but undergo surveillance endoscopy every 36 months and six months, respectively, according to the BSG recommendations on diagnosis and management of BE [13].

**Fig 1:**
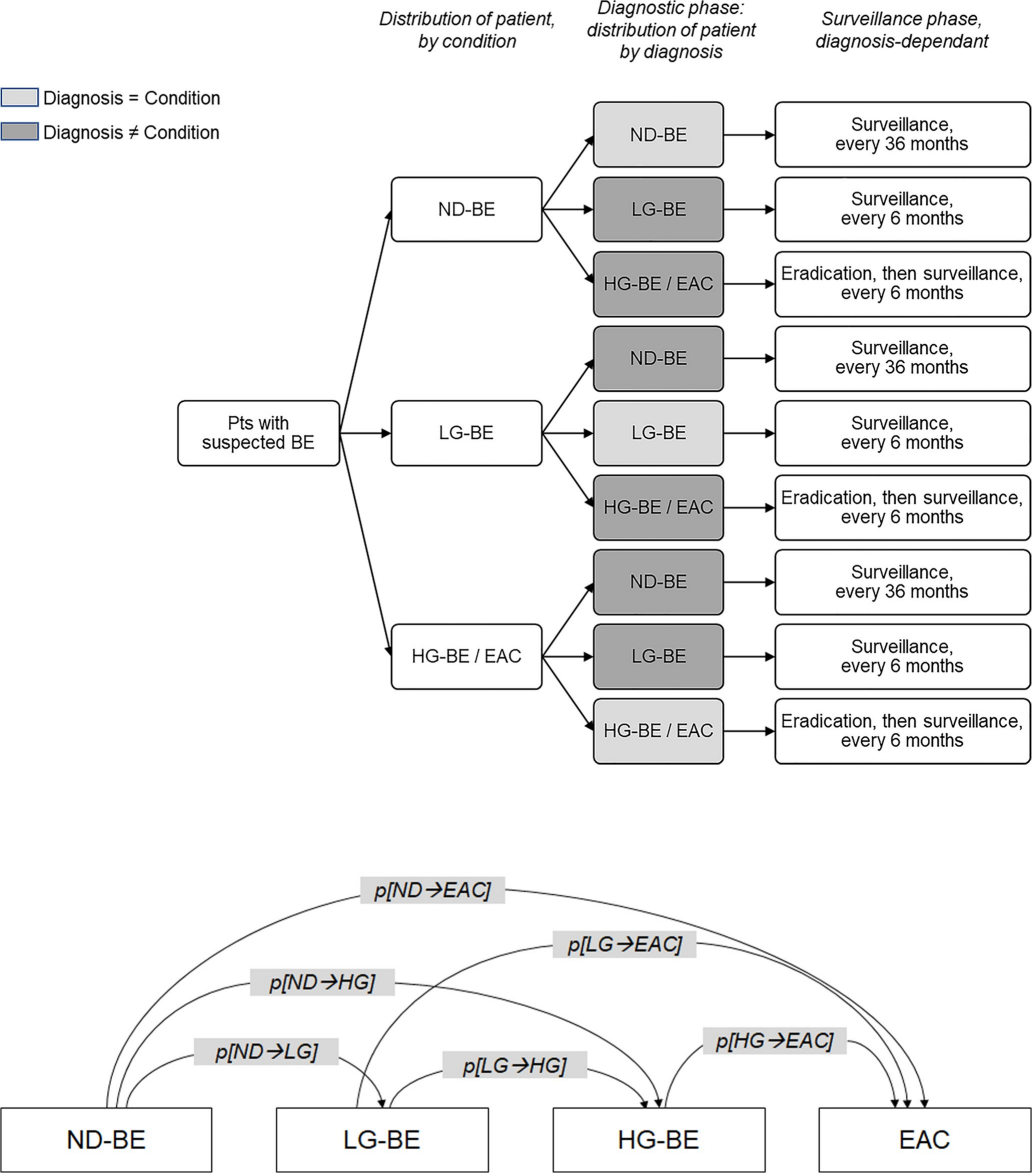
Decision tree and Markov model diagrams. (A) Decision tree diagram. (B) Markov model diagram. BE, Barrett’s esophagus; ND-BE, non-dysplastic Barrett’s esophagus; LG-BE, low-grade Barrett’s esophagus; HG-BE, high-grade Barrett’s esophagus; EAC, esophageal adenocarcinoma.

The decision tree used to schematise the diagnostic and surveillance phases of the analysis is shown in Fig 1A. During their follow-up, patients may experience disease progression, from ND-BE, to LG-BE, HG-BE and finally EAC. Disease progression was modelled using a 6-month cycle Markov model, as shown in Fig 1B. Diagnostic techniques associated with high performance rates reduce the erroneous diagnoses (e.g. missed dysplastic cases) and consequentially have the potential of reducing the cases of progression to HG-BE, and finally, carcinoma. The base-case analysis reported in this article evaluates the diagnostic phase only. Alternative scenarios have been analysed to evaluate the effects of surveillance and disease progression.

### Identification of relevant clinical inputs

Outputs from a literature review (conducted in May 2017) were used to identify relevant clinical inputs for the model. Four predefined Population, Intervention, Comparator, and Outcome (PICO) scenarios were queried in the search to address four key research questions: Q1) What is the epidemiological burden and prognosis associated with BE?; Q2) What is the key evidence (from clinical trials, observational studies, reviews, guidelines) associated with NBI or other alternative techniques in the diagnosis and treatment of BE?; Q3) What is the comparative sensitivity, specificity, treatment effectiveness of NBI vs alternative techniques?; Q4) What are the main economic implications of BE management with NBI (vs alternative techniques) in the diagnosis / treatment of BE?

A literature review was conducted using both EMBASE and PubMed / MEDLINE databases. Search limits were applied, including articles: i) in English language only; ii) humans only; iii) published since 2005 (to avoid outdated evidence that could bias the evaluation); iv) evaluating diagnostic accuracy in BE, esophageal neoplasia, esophageal dysplasia, EAC; analysing disease incidence, prevalence, survival, progression rates, other relevant patient outcomes. Articles were excluded if they were not original articles (e.g. commentaries, editorials, non-systematic reviews, etc.). Search terms used to conduct the review are reported in S1 Table.

Of the 2,106 articles retrieved, 658 passed the first screen, and 285 were included for data extraction (S2 Table). Table 1 illustrates the key evidence that was selected to inform model inputs.

**Table 1:** Key evidence extracted from Embase / PubMed literature review.

Key evidence	Reference	Study aim	Material and methods	Summary of key evidence used in the model				
Distribution of patients attending endoscopy test, by BE type	Parasa et al. 2017 [19]	Determine the risk of patients with BE for progression to HD-BE and EAC	• Longitudinal study in US and Netherlands • Data collection from 1985 to 2014 • Primary outcome: development of HD-BE or EAC (Kaplan-Meier method)	• Total patients = 2,697, minus one patient with no intestinal metaplasia N = 2,696 • N = 905 patients (33.6%) with dysplasia • Of the N = 905 patients: ○ N = 751 (83.0%) with LG-BE ○ N = 106 (11.7%) with HG-BE ○ N = 48 (5.3%) with EAC				
Diagnostic accuracy, per-patient	Thosani et al. 2016 [14]	Assess diagnostic performance of endoscopic real-time imaging of BE with advanced imaging technologies	• Meta-analysis calculating the pooled sensitivity, negative predictive value, and specificity for: ○ Chromoendoscopy by using acetic acid and methylene blue ○ Opto-digital chromoendoscopy by NBI ○ CLE	• NBI pooled sensitivity = 94.2% • NBI negative predictive value = 97.5% • NBI specificity = 94.4%				
Diagnostic accuracy, per-patient	Sharma et al. 2013 [17]	Compare HD-WLE and NBI for detection of IM and neoplasia in BE	• International, randomised, crossover trial • N = 123 patients enrolled • HD-WLE examination: four quadrant biopsies every 2 cm, plus targeted biopsies of visible lesions (Seattle protocol) • NBI examination: targeted biopsies	• NBI targeted biopsies can have the same IM detection rate as an HD-WLE examination with the Seattle protocol while requiring fewer biopsies (3.6 vs 7.6 respectively) • In addition, NBI targeted biopsies can detect more areas with dysplasia				
Diagnostic accuracy, per-lesion	Jayasekera et al. 2012 [20]	Assess the accuracy of predicting HD-BE and IMC as being nondysplastic vs. dysplastic with HD-WLE, NBI, and CLE	• Cross-sectional study • N = 50 consecutive patients (Feb 2010-Sep2011) with BE • N = 1,190 individual biopsy points assessed	• Per-location analysis of sensitivity and specificity for the detection of HD-BE and IMC • Sensitivity: HD-WLE = 79.1%; NBI = 89.0%; CLE = 75.7% • Specificity: HD-WLE = 81.0%; NBI = 80.0%; CLE = 75.7%				
Eradication rate and treatment-associated safety	Desai et al. 2017 [21]	Derive pooled rates of efficacy and safety of focal endomucosal resection followed by radiofrequency ablation (f-EMR + RFA) and stepwise or complete EMR (s-EMR)	• Systematic literature review in BE • Outcomes evaluated: ○ Complete eradication rates of neoplasia ○ Complete eradication rates of intestinal metaplasia ○ Recurrence rates of cancer, dysplasia and metaplasia ○ incidence rates of adverse events	• Rate of successful eradication: 94.9% • Incidence of adverse events: ○ Strictures = 33.5% ○ Perforations: 1.3% ○ Bleedings = 7.5%				
Disease progression rates	Shaheen et al. 2009 [22]	Assess the efficacy of RFA in eradicating dysplastic BE	• Multicenter, sham-controlled trial • N = 127 patients with dysplastic BE, randomised 2:1 (RFA: sham) • Primary outcomes at 12 months: complete eradication of dysplasia and intestinal metaplasia	6-month disease progression rates (elaborated)*				
					**ND-BE**	**LG-BE**	**HG-BE**	**EAC**
				**ND-BE**	0.9500	0.0500	0.0000	0.0000
				**LG-BE**	0.0000	0.9318	0.0682	0.0000
				**HG-BE**	0.0000	0.0000	0.9048	0.0952
				**EAC**	0.0000	0.0000	0.0000	1.0000
Diagnostic accuracy histopathology	Vennalaganti P et al. 2017 [15]	Assess the inter-observer agreement (among expert gastrointestinal pathologists) in the diagnosis of LG-BE in patients with BE	• Analysis of N = 79 histology slides from patients with BE (mixed sample of ND-BE, LG-BE, HG-BE), by N = 7 pathologists • Geographical scope: US (N = 4), EU (N = 3)	Estimated sensitivity and specificity of histopathology (elaborated*): • Sensitivity: 100.0% • Specificity: 62.9% *N = 14 cases who were classified as dysplastic cases in 1st assessment*, *were not in 2nd assessment (i*.*e*. *false positive)*.				

*Reported values are the result of elaboration of article data and findings.

BE, Barrett’s esophagus; HD-BE, high-grade dysplasia; EAC, esophageal adenocarcinoma; LG-BE, low-grade dysplasia. CLE, Confocal laser endomicroscopy; HD-WLE, high-definition white light endoscopy; IM, intestinal metaplasia; IMC, intramucosal cancer; f-EMR, focal endomucosal resection; RF, radiofrequency ablation; s-EMR, stepwise EMR; ND-BE, non-dysplastic Barrett’s esophagus; LG-BE, low-grade Barrett’s esophagus; HG-BE, high-grade Barrett’s esophagus; NBI, narrow-band imaging; Pt, patient.

### Clinical and cost inputs

A range of intervention-specific and non-specific clinical and cost inputs were used in the model and sourced from the published literature, NHS Reference Costs, and Olympus data on file (see Table 2). Both costs and outcomes after Year 1 were discounted at an annual rate of 3.5%, as recommended by the National Institute for Care and Clinical Excellence [23]. Costs were reported in GBP 2017. Cost inputs in other currencies (i.e. not GBP) were inflated using national inflation rates, then converted to GBP 2017.

**Table 2:** Default clinical and cost inputs.

Input	NBI	HD-WLE	Effect on model / analysis results	Source
Patients with known / suspected BE, attending endoscopy (n / year), NHS England	161,657		Patient population size influences national budget impact, as it determines the number of procedures that are performed annually at national level	England adult population (≥18 years; N = 43.1 mln [24] x BE prevalence (0.5% [3]) x diagnosis rate (75% [25])
Patients with known / suspected BE, attending endoscopy (n / year), Hospital perspective	649		Patient population size influences hospital budget impact, as it determines the number of procedures that are performed annually at national level	Total number of patients (N = 161,657), divided by NHS hospitals equipped to conduct Barrett’s surveillance (N = 249; 498 x 50%) i.e. 161,657 / 249 = 649 [25]
Annual increase in population attending for endoscopy (%)	20%		The increase in performed procedures determines an increase in the patient population size	Public Health England. 2013 [26] Health and Social Care Information Centre. 2012 [27]
Proportion of patients with nondysplastic BE (%)	66.4%		Distribution of patients is used to allocate patients in the first node of the decision tree (Fig 1A)	Elaboration from Parasa et al. 2017 [19]
Proportion of patients with dysplastic BE, low-grade (%)	83.0%			
Proportion of patients with dysplastic BE, high-grade (%)	11.7%			
Proportion of patients with EAC (%)	5.3%			
Sensitivity (%)	94.2%	94.2%	According to diagnostic accuracy, patients are allocated in the second node of the decision tree (Fig 1A)	For NBI, Thosani et al. 2016 [14]; *Assumption*: *same diagnostic accuracy*, *based on Sharma et al*. *2013* [17]
Specificity (%)	94.4%	94.4%		
Endoscopy: number of biopsies per intervention (n)	3.6	7.6	Number of biopsies performed influences costs and incidence of adverse events	Sharma et al. 2013 [17]
Endoscopy: incidence of strictures (%)	<0.01%	<0.01%	Occurrence of adverse events associated with diagnostic endoscopy determines additional management costs	British Society of Gastroenterology. 2018 [18] Ben-Menachem et al. 2012 [28] *Assumption*: *occurrence of post-procedural adverse events is rare*, *but still likely* [29]*; it decreases linearly with reduction of biopsies [Cause-consequence assumption based on external medical expert opinion]*
Endoscopy: incidence of perforations (%)	1.4%	3.0%		
Endoscopy: incidence of bleedings (%)	0.2%	0.5%		
Treatment: dysplasia eradication rate (%)	94.9%	94.9%	Patients with successfully eradicated dysplasia have lower risk of developing high-grade dysplasia or carcinoma vs non-eradicated and generate lower costs	Desai et al. 2017 [21] *Assumption*: *same efficacy and safety of NBI and HD-WLE*, *irrespective of diagnosis technique*
Treatment: incidence of strictures (%)	33.5%	33.5%	Occurrence of adverse events associated with dysplasia eradication determine additional management costs	
Treatment: incidence of perforations (%)	1.3%	1.3%		
Treatment: incidence of bleedings (%)	7.5%	7.5%		
Number of hospitals providing endoscopy (n)	249 (50% x 498)		Hospital data (number of hospitals, rooms, endoscopists, scopes) are used to calculate costs to fully equip hospitals to perform endoscopies and treatment of dysplasia cases	Internal market data Equipment amortization: UK Department of Health Depreciation Policy for Primary Care Trusts [30]
Average number of endoscopy rooms per hospital (n)	3.25			
Average number of endoscopists per hospital (n)	3.25			
Average number of scopes per endoscopy room	4.0			
Technique market share (%)	84%	100%	Market shares indicate the adoption of each technique	
Proportion of NBI-capable systems already in place (%)	83.0%	100.0%	Market data (% of capable systems, % of already purchased scopes, etc.) are used to calculate costs to fully equip hospitals for performing endoscopies and treatment of dysplasia cases	
Proportion of HD scopes already in place (%)	40.0%	100.0%		
Proportion of endoscopists already trained (%)	50.0%	100.0%		
Proportion of old scopes to replace, per year (%)	0.0%	2.9%		
Capital equipment: unit cost per system (£)	41,316	41,316	Capital equipment data are used to calculate the annual costs that hospital incur to purchase new instrumentation, maintain the existing one, and train physicians on the use of the most updated technologies. Costs depend on the proportion of equipment to be purchased, vs proportion of equipment to be maintained and / or replaced. Investment for purchased equipment is distributed over 7 years	
Capital equipment: unit cost per scope (£)	30,487	30,487		
Capital equipment: training cost (£/day)	1,136	795		
Capital equipment: training days per endoscopist (n)	2	0		
Capital equipment: maintenance cost, system (£/year)	4,590	4,527		
Capital equipment: maintenance cost, scopes (£/year)	4,285	4,089		
Capital equipment: time to amortization (years)	7			
Staff cost: administration, before/after endoscopy (£/hour)	23		Hourly staff costs are multiplied by procedural time to calculate personnel costs to execute endoscopy and dysplasia treatment	Personal Social Services Research Unit. 2014 [31]
Staff cost: nurse non-contact, before/after endoscopy (£/hour)	41			
Staff cost: consultant contact, before/after endoscopy (£/hour)	142			
Staff cost: nurse contact, during endoscopy (£/hour)	100			
Staff cost: consultant contact time, during endoscopy (£/hour)	142			
Staff time: administration, before/after endoscopy (hrs)	0.30	0.30	Procedural time is multiplied by hourly staff costs to calculate personnel costs to execute endoscopy and dysplasia treatment	Sharara et al. 2008 [32] *Assumption*: *nurse non-contact time is proportional to the number of biopsies*
Staff time: nurse non-contact, before/after endoscopy (hrs)	0.42	0.89		
Staff time: consultant contact, before/after endoscopy (hrs)	0.50	0.50		
Staff time: nurse contact, during endoscopy (hrs)	0.30	0.30		
Staff time: consultant contact time, during endoscopy (hrs)	0.30	0.30		
Consumable cost: snares, 20 units per pack (£)	240		Consumable costs are considered to calculate hospital costs for the execution of endoscopy and dysplasia eradication	Internal market data
Consumable cost: forceps, 10 units per pack (£)	210			
NHS tariff for esophageal endoscopy (£)	517		In analyses adopting the NHS perspective, these unit costs are used to calculate the overall costs to execute all endoscopies and dysplasia eradication, at national level	Code FZ03A [33]
NHS tariff for endomucosal resection + radiofrequency ablation (£)	2,101			Codes FZ24C, FZ24B, FZ24C, FZ24A [33]
Cost per biopsy (£)	82		Unit cost of biopsy is multiplied by the number of biopsies to calculate the cost associated with histological exams. Such cost is proportional to the number of biopsies per intervention	University College London Hospitals. 2012. [34] Plymouth Hospital NHS Trust. 2012. [35] South Devon Healthcare NHS Foundation Trust. 2014. [36]
Cost per histological exam (£)	295.2	623.2		Calculated: unit cost x number of biopsies per intervention
Cost of stricture (£)	392		Unit cost of adverse event management is multiplied by the adverse event rates to calculate the economic burden of adverse events. Such economic burden is finally proportional to the number of executed biopsies	Public Health England. 2013 [26]
Cost of bleeding (£)	392			Public Health England. 2013 [26]
Cost of perforation (£)	2,852			Health and Social Care Information Centre. 2012 [27]
Yearly cost of cancer management (£)	7,647		In the sensitivity analyses, considering long-term consequences, this annual cost Is multiplied by the number of adenocarcinoma cases. Occurrence of cancer cases depend on progression rates, as shown in Fig 1B	Elaborated from Gordon et al. 2011 [37]

BE, Barrett’s esophagus; EA, esophageal adenocarcinoma; Hrs, hours; HD-WLE, high-definition white light endoscopy; NHS, National Health Service; NBI, narrow-band imaging.

### Base-case analysis assumptions

The base-case analysis was conducted adopting the NHS England perspective and a time horizon of 7 years. In this analysis, the diagnostic performance of NBI and HD-WLE was based on per-patient level data, according to which NBI has the same dysplasia detection rate as an HD-WLE examination with the Seattle protocol, while requiring fewer biopsies [17].

The base-case considers that more than 160,000 patients with known or suspected BE undergo an endoscopic test in England and that this number is rapidly growing at a rate of 20% every year (references in Table 2).

The following input and assumptions were used to calculate capital equipment costs: 50% of total hospitals in England (N = 249; 498 x 50%) are equipped to conduct Barrett’s Esophagus surveillance endoscopy [25]; an average of 3.25 endoscopy rooms each and an average of 3.25 endoscopists per hospital [25]; 84% of the installed endoscopy systems were manufactured by Olympus; 83% of hospitals using Olympus endoscopy platforms were assumed to have NBI-capable systems in place; 40% of the available gastroscopes would be high-definition (HD) scopes that allow optical diagnosis using NBI; each endoscopy room is equipped with an average of four scopes. In the NBI scenario, it was plausibly assumed that all hospitals with installed endoscopy systems manufactured by Olympus would be equipped with NBI, while the remaining 16% would continue to adopt HD-WLE. No capital equipment investments were considered in the HD-WLE scenario, except for scope replacement due to depletion/damage (20% replacement over 7 years: 2.9%/year). It was considered that 50% of endoscopists who would adopt the NBI technique would also require two days of ad-hoc training based on training programmes provided by the manufacturer. Capital equipment costs have been amortized over the entire time horizon (7 years) in line with the average lifetime of the equipment and according to UK Department of Health Depreciation Policy for Primary Care Trusts [30]. The estimated 7 years takes into account both the system and scope lifetime; the system could be considered to have a 10 year i.e. medium life medical equipment, while the scope could be considered to have a 5 year life i.e. short life medical and other equipment [30]. Furthermore, the scope lifetime is highly dependent on the number of examinations, reprocessing method and handling, and ranges between 5–10 years based on internal service data.

### Scenario analysis

A range of analyses were conducted to inform on various analysis settings. On top of the base-case, five additional analyses were run by varying the following parameters: i) dysplasia diagnostic effectiveness (measured using per-patient data or per-lesion data); ii) perspective (NHS vs hospital perspective); iii) patient follow-up (not considering BE long-term consequences, i.e. cancer occurrence, vs. capturing BE long-term consequences, only applicable to NHS perspective).

In the hospital perspective, a micro-costing approach is used for calculation of endoscopic costs. The model defaults to assuming all resources are included as a cost with the exception of tariffs for endoscopy and treatment (i.e. EMR+RFA) which are included as reimbursements and thus considered income. In the NHS perspective, a micro-costing + tariff approach is used for calculation of endoscopic costs. The model defaults to assuming scopes and systems are direct costs to the NHS, in addition to the cost of tariffs for endoscopy and treatment; all other cost items are not applicable as they are covered under tariff. The number N = 649 corresponds to the estimated number of patients with known BE attending endoscopy in each UK hospital which is equipped to conduct BE surveillance.

### Sensitivity analysis

A deterministic, one-way sensitivity analysis (OWSA) was conducted on the results of the base-case cost-consequence analysis to quantify any uncertainty in results. Input data tested included demographics (number of patients with known or suspected BE, undergoing endoscopy), efficacy and safety (e.g. sensitivity and specificity of the two alternatives, rate of adverse events), and costs (e.g. capital equipment, biopsy). The lower and upper limits of all inputs were relative variations of ±10%, except for discounting costs (lower limit: 2.0%; upper limit: 5.0%) and diagnostic accuracy (lower and upper limits as per 95% confidence intervals reported in the study [14]). One additional sensitivity analysis was conducted, assuming that adverse event rates (for bleedings and perforations) would not depend on the number of executed biopsies (i.e. same adverse event rate for NBI and HD-WLE).

## Results

### Base-case analysis

Table 3 shows the results of the cost-consequence analysis in the base-case scenario (i.e. NHS perspective; time horizon: 7 years; diagnostic effectiveness based on per-patient specificity / sensitivity). After 7 years, the adoption of NBI optical imaging (with targeted biopsies) resulted in cost reduction of £458.0 mln, vs standard approach (HD-WLE with random biopsies). The incremental investment on capital equipment to upgrade hospitals with NBI technology (+£68.3 mln) was entirely offset by savings, primarily due to the reduction of histological examination (-£505.2 mln). Reduction of biopsies per intervention also determined savings for avoided adverse events (-£21.1 mln).

**Table 3:** Base-case: Results of the cost-consequence analysis (time horizon: 7 years).

Category	NBI	HD-WLE	Absolute difference, NBI vs HD-WLE	Relative difference, NBI vs HD-WLE
*Costs*				
Endoscopy, staff and overheads (£mln)	948.1	948.1	0.0	0.0%
Treatment, staff and overheads (£mln)	214.3	214.3	0.0	0.0%
Histopathology, costs (£mln)	637.6	1,142.8	-505.2	-44.2%
Adverse events (£mln)	62.4	83.4	-21.1	-25.3%
Capital equipment (£mln)	103.9	35.6	68.3	192.2%
**Totals costs (£mln)**	**1,966.2**	**2,424.2**	**-458.0**	**-18.9%**
*Outcomes*				
Number of correctly identified cases (n)	1,934,602	1,934,602	0.0	0.0%
Number of successful eradications (n)	77,331	77,331	0.0	0.0%
Number of biopsies (n)	8,852,892	15,868,392	-7,015,500	-44.2%
Number of AEs: strictures (n)	21,092	21,092	0	0.0%
Number of AEs: bleedings (n)	36,456	64,149	-27,693	-43.2%
Number of AEs: perforations (n)	14,537	19,152	-4,615	-24.1%

AEs, adverse events; HD-WLE, high-definition white light endoscopy; NBI, narrow-band imaging.

In the budget impact analysis, the estimated annual savings were shown to increase each year as the population attending for surveillance endoscopy continued to grow (Table 4). By year 7, the use of NBI was associated with annual cost savings to NHS England of over £103.1 mln.

**Table 4:** Base-case: Results of the budget impact analysis.

Year	Population undergoing esophageal endoscopy (n)	NBI (£mln)	HD-WLE (£mln)	Absolute difference, NBI vs HD-WLE (£mln)
1	161,657	180.6	216.2	-35.6
2	193,988	206.0	249.3	-43.3
3	232,786	235.5	287.6	-52.1
4	279,343	269.7	332.1	-62.3
5	335,212	309.5	383.5	-74.0
6	402,254	355.6	443.2	-87.5
7	482,705	409.2	512.3	-103.1
**Total**	**2,087,946**	**1,966.2**	**2,424.2**	**-458.0**

HD-WLE, high-definition white light endoscopy; NBI, narrow-band imaging.

Cost-effectiveness analysis was not applicable in the base-case analysis as it was assumed that NBI and HD-WLE had the same diagnostic performance based on per-patient sensitivity and specificity. This conservative approach was adopted in order not to overstate the benefit of NBI, considering that: i) the primary evidence used in the model from Sharma et al [17] concluded that NBI is as effective as HD-WLE but safer; ii) other evidence showed superior comparable diagnostic accuracy of NBI vs HD-WLE [38,39]. Therefore, the two techniques resulted in an identical number of correctly identified dysplasia cases (N = 1.935 mln) and the same number of successful eradications (N = 77,331). However, compared with HD-WLE, NBI substantially reduced the number of taken biopsies (-7.016 mln) and burden of adverse events (relative reduction for all adverse events: -30.9%).

### Scenario analysis

Results of alternative scenarios in the NHS or hospital perspective are summarized in Table 5. Each conducted analysis showed that the adoption of NBI would be cost-saving in those scenarios where diagnostic accuracy was measured on a per-patient level (NBI and HD-WLE have same detection rate [17]), and dominant in those scenarios where diagnostic accuracy was measured on a per-lesion level (NBI has higher detection rate than HD-WLE [20]). In the scenario where patients were followed-up for 7 years (extended follow-up to capture occurrence of cancer cases) and where diagnostic effectiveness was measured on a per-lesion level suggested a potential moderate benefit of NBI in reducing the burden of cancer incidence (N = 1,615 cases avoided over 161,657 observed patients). As NBI was more effective than HD-WLE in detecting dysplastic lesions, it reduced the number of false negative patients who would otherwise be undiagnosed and at a high risk of esophageal cancer progression. However, in these scenarios, the potential reduction in cancer prevention was somewhat mitigated by the fact that all patients who were initially undiagnosed underwent surveillance, and therefore could be correctly identified during follow-up.

**Table 5:** Scenario analysis: Results of the cost-consequence analysis from the NHS and hospital perspectives (time horizon: 7 years).

Scenario number	Scenario description	Results of the cost-consequence analysis	Results of the cost-effectiveness analysis
**NHS perspective (N = 161,657)**			
1	• Diagnostic performance: per-patient • Patient follow-up: BE-long term consequences not considered	NBI cost saving vs HD-WLE: £458.0 mln savings	Same effectiveness, NBI cost-saving
2	• Diagnostic performance: per-patient • Patient follow-up: BE-long term consequences considered	NBI cost saving vs HD-WLE: £349.2 mln savings	Same effectiveness, NBI cost-saving
3	• Diagnostic performance: per-lesion • Patient follow-up: BE-long term consequences not considered	NBI cost saving vs HD-WLE: £417.0 mln savings	NBI dominates HD-WLE (£417.0 mln savings; +30,727 detected lesions; ICER: -£13.5K per incremental detected lesion)
4	• Diagnostic performance: per-lesion • Patient follow-up: BE-long term consequences considered	NBI cost saving vs HD-WLE: £331.5 mln savings	NBI dominates HD-WLE (£331.5 mln savings; 1,615 cancer cases avoided; ICER: -£205K per avoided cancer case)
**Hospital perspective (N = 649)**			
5	• Diagnostic performance: per-patient • Patient follow-up: BE-long term consequences not considered	NBI cost saving vs HD-WLE: £2.5 mln savings; NBI more marginal than HD-WLE: +£2.5 mln	Not applicable (NBI as effective as HD-WLE)
6	• Diagnostic performance: per-lesion • Patient follow-up: BE-long term consequences not considered	NBI cost saving vs HD-WLE: £2.5 mln savings; NBI more marginal than HD-WLE: +£2.6 mln	NBI dominates HD-WLE (£11.7 mln margin; +622 detected lesions; ICER: -£18.8K per incremental detected lesion)

HD-WLE, high-definition white light endoscopy; NBI, narrow-band imaging; NHS, National Health Service; ICER, incremental cost-effectiveness ratio

### Sensitivity analysis

**Fig 2:**
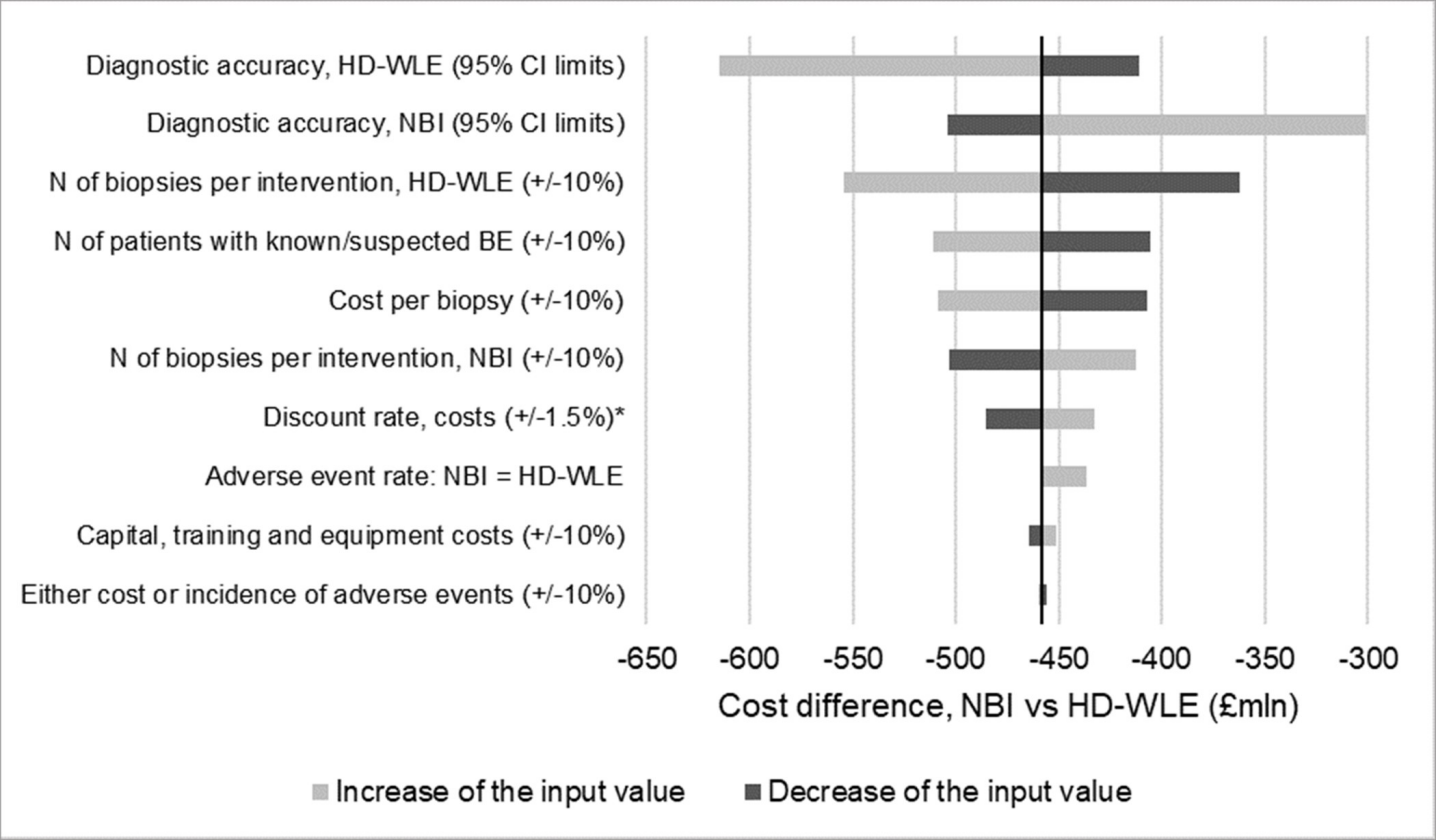
Results of the deterministic one-way sensitivity analysis. *Absolute change of the input value. BE, Barrett’s esophagus; CI, confidence interval; HD-WLE, high-definition white light endoscopy; NBI, narrow-band imaging.

OWSA confirmed robustness of the base-case analysis findings (Fig 2). Cost-saving associated with NBI adoption was confirmed in all analyses. As expected, the input parameters associated with biopsy demonstrated the largest effect on the outputs, which was the cost driver of the analysis. However, even the worst case identified by the deterministic sensitivity analysis (DSA; lower 95% confidence interval of the diagnostic accuracy of NBI) would result in savings of £301.0 mln with NBI. A -10% decrease in the number of biopsies per intervention compared with HD-WLE also had an impact on the outputs; however, would result in savings of £362.0 mln with NBI, plus a potential benefit in terms of number of identified dysplasia cases (a reduction in the number of biopsies per intervention would result in inappropriate execution of the Seattle protocol, and a consequent loss in detection rate). NBI was still cost-saving vs HD-WLE, under the assumption of same adverse event rate in the two groups (-£436.9 mln).

## Discussion

BE is a common condition, characterized by negative impact on patient prognosis and quality of life, plus significant economic burden for healthcare services. According to epidemiology estimates, there are at least 160,000 patients with BE in England (Table 2). This number may be underestimated, as a low-end prevalence assumption was applied (0.5%; range: 0.5%-2.0%), as well as a 75% diagnosis rate to take into account that: i) BE may be underdiagnosed; ii) a certain proportion of patients do not receive care during the year. Therefore, it is plausible that the BE population in England will increase over the coming years to be in the range of 250,000 to 300,000 interventions per year.

The annual budget impact associated with BE is significant. In our analysis, per-patient costs and Year-1 budget impact (in the HD-WLE, Seattle protocol group) were approximately £1,340 and £216 mln, respectively (Table 4). Two components drove such healthcare expenditure and accounted for more than 90% of overall costs: i) hospital remuneration for both endoscopic and eradication procedures (£102 mln: 161,000 endoscopic procedures, times £517 per endoscopy, plus 9,000 eradications, times £2,100 per eradication; per-patient cost: £634); ii) histopathology costs (£101 mln: 161,000 endoscopic procedures, times 7.6 biopsies per endoscopy, times £82 per biopsy; per-patient cost: £623). If this economic impact is evaluated over a 7-year period (assuming that the number of procedures is destined to increase), costs for NHS England would be in the magnitude of £2.4 billion. In contrast, NBI has the potential to reduce per-patient costs and Year-1 budget impact to approximately £1,120 (cost-saving vs HD-WLE: £220) and £181 mln (cost-saving vs HD-WLE: £35.6 mln), respectively. In the NBI group, costs of hospital remuneration for both endoscopic and eradication procedures were the same as in the HD-WLE group, while histopathology costs decrease substantially (overall costs: £56 mln: per-patient costs: £348). Since a reduction in the number of annual procedures is unlikely in the future, reduction of the economic burden of histopathology is a plausible option to mitigate costs, reduce NHS operators’ workload, and ideally re-allocate efficiency savings. The economic comparison of NBI with targeted biopsies vs HD-WLE with random biopsies relies on the assumption that the two alternatives are equally effective, while the targeted approach reduces the number of executed biopsies by at least 50%. From a clinical perspective, the targeted approach is also safer and more conservative for patients, who are exposed to a lower risk of treatment-related adverse events.

Our literature review confirms this hypothesis: recent meta-analyses from renowned scientific associations [14] have endorsed the use of advanced imaging modalities to guide targeted biopsies for the detection of dysplasia during surveillance of patients with BE, acknowledging they have the potential to replace currently used random biopsy protocols. Although other techniques like electronic chromoendoscopy (e.g. supported by i-SCAN, Pentax Medical, and FICE or Fujinon Intelligent Chromo Endoscopy, Fujinon Inc.) have evolved, evidence associated with the use of opto-digital chromoendoscopy with NBI remains one of the most compelling [14,40].

Nevertheless, given the developments in this therapeutic area, regular surveillance of the literature is strongly recommended to ensure the most recent and appropriate sources are captured in the economic analysis.

Although every effort was made to ensure robustness of the model and its results, the economic model has several limitations. First, due to the lack of clear data regarding the annual number of esophageal procedures executed by NHS England, estimates on the number of BE patients were based on epidemiological calculations (i.e. England adult population x BE prevalence x diagnosis rate). This approach was adopted since NHS reports grouped esophageal procedures together with those involving the upper gastro-intestinal tract. For this reason, it was not possible to determine the number of esophageal procedures from NHS activity reports. Conservatively, we adopted low-end estimates for BE prevalence and applied an arbitrary reduction factor of 25% to take into account that BE might be under-diagnosed. As a result, budget impact estimates in the first years of analysis could have been underestimated. Regardless, conclusions of the cost-consequence analysis (i.e. NBI cost saving vs HD-WLE) would not be affected by such underestimation, thus maintaining validity.

Second, we assumed no difference in detection rates between NBI and WLE, which may not be reflective of real-life clinical practice. This assumption was based on a single study [17], but was also confirmed by a recent meta-analysis highlighting the validity of using advanced imaging to guide targeted biopsies [14]. Again, we preferred to adopt a conservative approach for NBI, although some evidence suggests that NBI might be even more accurate than standard of care in detecting dysplasia [20].

Moreover, there was uncertainty regarding the level of adherence to the Seattle Protocol, which may not be universally followed by endoscopists [41,42]. Although the economic model was developed to potentially adjust sensitivity and specificity of the Seattle Protocol by suboptimal execution of biopsies, we did not present results of this sensitivity analysis, as the magnitude of dysplasia detection rate loss (if lower-than-recommended biopsies are executed) could not be identified in the literature. Plausibly, this analysis would have resulted in a reduction of the cost saving effect associated with NBI usage, but in overall better clinical outcomes, compared with the HD-WLE group (i.e. NBI dominant vs HD-WLE).

Third, there were limitations around reliability in some of cost inputs incorporated. While remuneration items (e.g. NHS tariffs for esophageal endoscopy, NHS tariffs for endomucosal resection and radiofrequency ablation) should realistically reflect the economic expenditure for NHS England, costs of histological exams might be affected by some variability (no NHS costs were identified, input data were sourced from a similar article [43], which used an average cost calculated from three private and provider-to-provider prices lists [34–36]. Additionally, the cost per biopsy incurred by hospitals and how this is passed through to the NHS is expected to vary by hospital.

Finally, market share assumptions (in terms of the proportion of hospitals currently using NBI-capable equipment or likely to upgrade if this strategy was recommended) were sourced from internal market data and may not, therefore, entirely reflect current market trends. However, the uncertainty associated with resource consumption and unit cost assumptions was tested through sensitivity analysis, which confirmed findings of the base-case analysis.

## Conclusions

The results of our model suggest that NBI with targeted biopsies is a cost-saving strategy for NHS England compared to current practice, i.e. HD-WLE with random biopsies, for the detection of dysplasia in patients with BE, and may ensure at least comparable health outcomes for patients. However, since the constant evolution of diagnostic techniques will likely determine further improvements in the diagnosis and treatment of BE, it is highly recommended to review and update the available evidence, and conduct analyses reflecting such developments, including the selection of new comparators.

## Supporting information

S1 Table String search approach used for Embase / PubMed literature review. (DOCX)

S2 Table Embase / PubMed literature review findings. (DOCX)
