# Supplementary material for: Economic value of narrow-band imaging versus white light endoscopy for the diagnosis and surveillance of Barrett’s esophagus: Cost-consequence model
Source: PLoS One. 2019 Mar 13;14(3):e0212916. doi: 10.1371/journal.pone.0212916 (PMC6415878; doi:10.1371/journal.pone.0212916)
Supplement: S1 Table — (DOCX) [file pone.0212916.s001.docx]

**S 1 Table.** **String search approach used for Embase / PubMed literature review**

| **Question** | **String search** |
| --- | --- |
| Q1) What is the epidemiological burden and the prognosis associated with BE? | (Barrett’s Esophagus) AND (incidence OR prevalence) AND mortality |
| Q2) What is the key-evidence (from clinical trials, observational studies, reviews, guidelines) associated with NBI or other alternative techniques in the diagnosis / treatment of BE? | (Barrett’s Esophagus) AND (Narrow Band Imaging OR NBI OR Conventional endoscopy OR white light endoscopy OR white-light endoscopy OR WLE OR white light imaging OR white-light imaging OR WLI OR conventional chromoendoscopy OR virtual chromoendoscopy OR Fuji Intelligent Colour Enhancement OR FICE OR i-Scan OR iScan OR BLI OR Blue laser Imaging OR OE OR Optical enhancement OR confocal laser endomicroscopy OR CLE OR plasma coagulation or endoscopic radiofrequency or endoscopic eradication or non-biopsy) AND (study OR trial OR Diagnosis OR management OR diagnostic guideline OR clinical guidance OR clinical guideline) |
| Q3) What is the comparative sensitivity, specificity, treatment effectiveness of NBI vs other alternative techniques? | ((Barrett’s Esophagus) AND (Narrow Band Imaging OR NBI) AND (Conventional endoscopy OR white light endoscopy OR white-light endoscopy OR WLE OR white light imaging OR white-light imaging OR WLI OR conventional chromoendoscopy OR virtual chromoendoscopy OR Fuji Intelligent Colour Enhancement OR FICE OR i-Scan OR iScan OR BLI OR Blue laser Imaging OR OE OR Optical enhancement OR confocal laser endomicroscopy OR CLE OR plasma coagulation or endoscopic radiofrequency or endoscopic eradication or non-biopsy)) AND (((Accuracy OR Sensitivity OR Specificity OR NPV OR Feasibility OR Confidence OR Inter-observer agreement) OR (Safety OR complications OR adverse events)) OR Training) |
| Q4) What are the main economic implications of BE management with NBI (vs alternative techniques) in the diagnosis / treatment of BE? | (Barrett’s Esophagus) AND ((Narrow Band Imaging OR NBI) AND (Conventional endoscopy OR white light endoscopy OR white-light endoscopy OR WLE OR white light imaging OR white-light imaging OR WLI OR conventional chromoendoscopy OR virtual chromoendoscopy OR Fuji Intelligent Colour Enhancement OR FICE OR i-Scan OR iScan OR BLI OR Blue laser Imaging OR OE OR Optical enhancement OR confocal laser endomicroscopy OR CLE OR plasma coagulation or endoscopic radiofrequency or endoscopic eradication or non-biopsy)) AND ((Cost* OR economic OR budget OR cost benefit OR cost effectiveness* OR cost minimization OR cost utility OR economic evaluation OR price* OR health technology assessment OR HTA) OR (Resource use OR utilization)) |
