# Supplementary material for: Economic value of narrow-band imaging versus white light endoscopy for the diagnosis and surveillance of Barrett’s esophagus: Cost-consequence model
Source: PLoS One. 2019 Mar 13;14(3):e0212916. doi: 10.1371/journal.pone.0212916 (PMC6415878; doi:10.1371/journal.pone.0212916)
Supplement: S2 Table — (DOCX) [file pone.0212916.s002.docx]

**S 2 Table. Embase / PubMed literature review findings**

|  | **String search** | | | | |
| --- | --- | --- | --- | --- | --- |
|  | **Q1** | **Q2** | **Q3** | **Q4** | **All strings** |
| **PubMed search, N** | **348** | **856** | **49** | **12** | **1,265** |
| **Embase search, N** | **279** | **1006** | **109** | **28** | **1,422** |
| **Search w/o duplicates, N** | **516** | **1,423** | **133** | **34** | **2,106** |
| **Articles passing the title/abstract screening, N** *[reasons for exclusion]* | **210**  *[N=306 articles excluded: published before 2005 (n=97); topic not in scope (n=71); not original article / letter / commentary (n=136); other (n=2; 1 study in animals; 1 study with small sample size)]* | **390**  *[N=1,033 articles excluded: published before 2005 (n=114); topic not in scope (n=141); not original article / letter / commentary (n=755); other (n=23; 9 studies in animals; 13 studies with small sample size or case reports; 1 study without relevant results)]* | **50**  *[N=83 articles excluded: published before 2005 (n=1);  topic not in scope (n=2); not original article / letter / commentary (n=80)]* | **8**  *[N=26 articles excluded: topic not in scope (n=1);  not original article / letter / commentary (n=25)]* | **658** |
| **Articles passing the full text screening**  *[reasons for exclusion]* | **81**  *[N=129 articles excluded: topic not in scope (n=46); not original article / letter / commentary (n=83)]* | **202**  *[N=188 articles excluded: topic not in scope (n=95); not original article / letter / commentary (n=83); other (n=10; 1 study in animals; 6 studies with small sample size; 2 studies without relevant results),  1 repeated article)]* | **32**  *[N=18 articles excluded: not in scope / non-comparative design (n=5); not original article / letter / commentary (n=13)]* | **2**  *[N=6 articles excluded: not original article /  letter / commentary (n=6)]* | **285**  *[N=317 articles, minus 34 duplicate articles, extracted in more than 1 string search, plus 2 correlated articles cited by extracted articles and rated as key-evidence]* |
